# Supplementary material for: Cholesterol-induced LRP3 downregulation promotes cartilage degeneration in osteoarthritis by targeting Syndecan-4
Source: Nat Commun. 2022 Nov 21;13:7139. doi: 10.1038/s41467-022-34830-4 (PMC9681739; doi:10.1038/s41467-022-34830-4)
Supplement: Supplementary file 1 — Supplementary Information [file 41467_2022_34830_MOESM1_ESM.pdf]

Supplementary figure 1

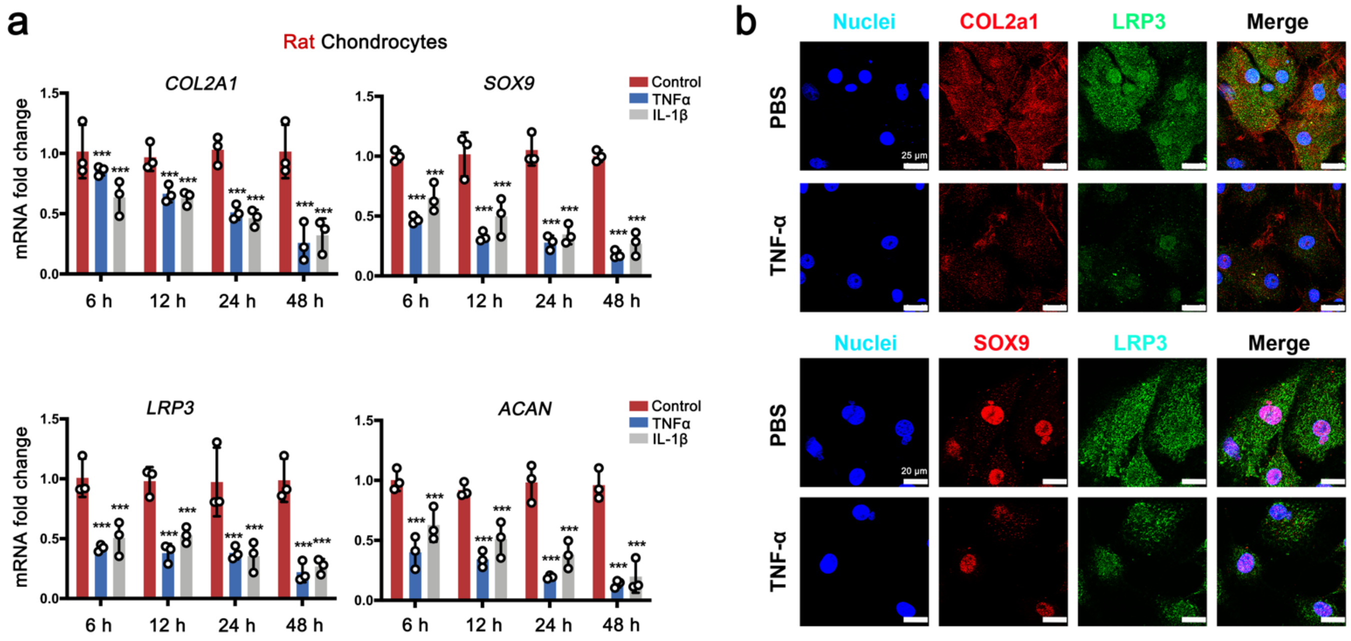

Supplementary figure 1 a Quantification of mRNA levels for *COL2A1*, *SOX9*, *LRP3* and *ACAN* in rat chondrocytes treated with TNF-α (20 ng/ml) or IL-1β (10 ng/ml) at different times (n = 3, one-way ANOVA). **b** Immunofluorescence co-staining for COL2A1, SOX9 and LRP3 in rat chondrocytes treated with TNF-α (20 ng/ml). Data are shown as the mean ± SD. **P* < 0.05; ***P* < 0.01; ****P* < 0.001. n indicates the number of biologically independent samples.

Supplementary figure 2

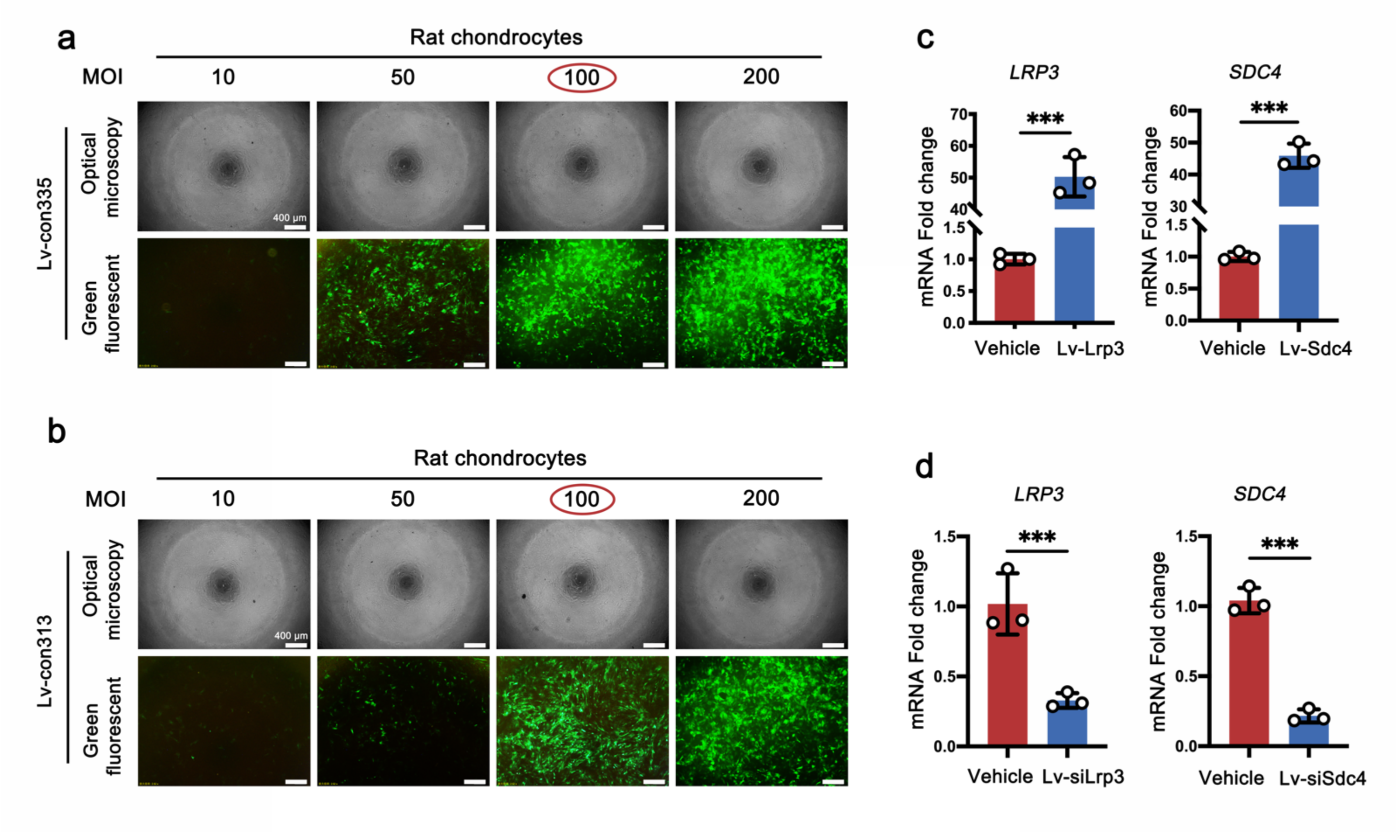

Supplementary figure 2 a Fluorescence image of rat chondrocytes infected with different MOI overexpression lentivirus empty vector. **b** Fluorescence image of rat chondrocytes infected with different MOI knockdown lentivirus empty vector. **c** Quantification of mRNA levels for *Lrp3* and *Sdc4* in rat chondrocytes infected with overexpression lentivirus under 100 MOI (n = 3, two-tailed Student's *t*-test). **d** Quantification of mRNA levels for *Lrp3* and *Sdc4* in rat chondrocytes infected with knockdown lentivirus under 100 MOI (n = 3, two-tailed Student's *t*-test). Data are shown as the mean ± SD. **P* < 0.05; ***P* < 0.01; ****P* < 0.001. n indicates the number of biologically independent samples.

Supplementary figure 3

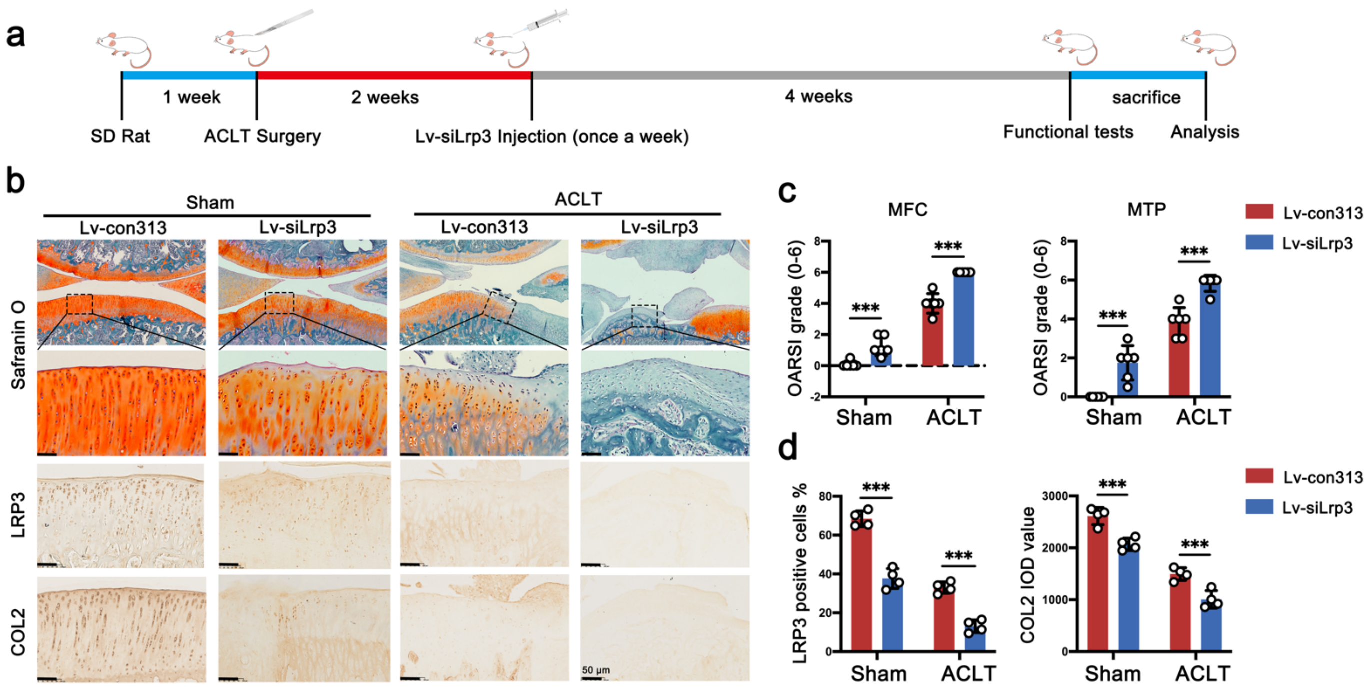

Supplementary figure 3 a Scheme of ACLT surgery and Lv-siLrp3 injection in rats. **b** Representative images of safranin O-fast green (n = 6), LRP3 and COL2 IHC staining (n = 4) of knee joints from Lv-siLrp3 injected rats and Lv-con313 injected rats subjected to Sham or ACLT operation for 4 weeks, insets indicate the regions shown in the enlarged images. **c** OARSI scores of Lv-siLrp3 injected rats and Lv-con313 injected rats subjected to Sham or ACLT operation for 4 weeks (n = 6, two-tailed Student's *t*-test). **d** Quantification of LRP3-positive cells and IOD value for COL2 in Lv-siLrp3 injected rats and Lv-con313 injected rats subjected to Sham or ACLT operation for 4 weeks (n = 4, two-tailed Student's *t*-test). Data are shown as the mean ± SD. $^{*}P < 0.05$; $^{**}P < 0.01$; $^{***}P < 0.001$. n indicates the number of biologically independent samples or mice per group.

Supplementary figure 4

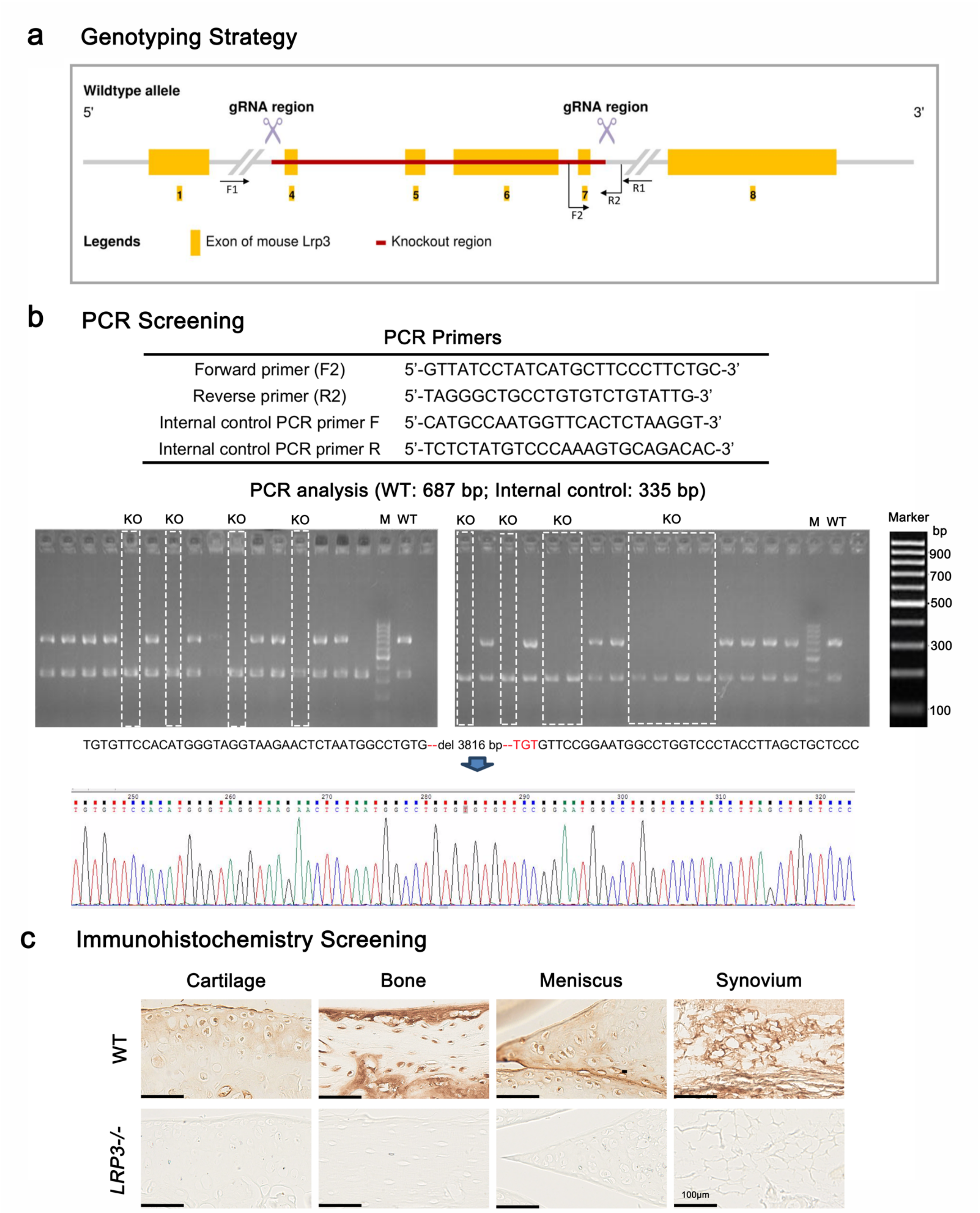

Supplementary figure 4 a The genotyping strategy of *Lrp3*$^{-/-}$ mice construction. **b** The PCR screening of LRP3$^{-/-}$ mice. **c** The IHC staining for LRP3 in cartilage, bone, meniscus and synovium of WT or *Lrp3*$^{-/-}$ mice (n = 3).

Supplementary figure 5

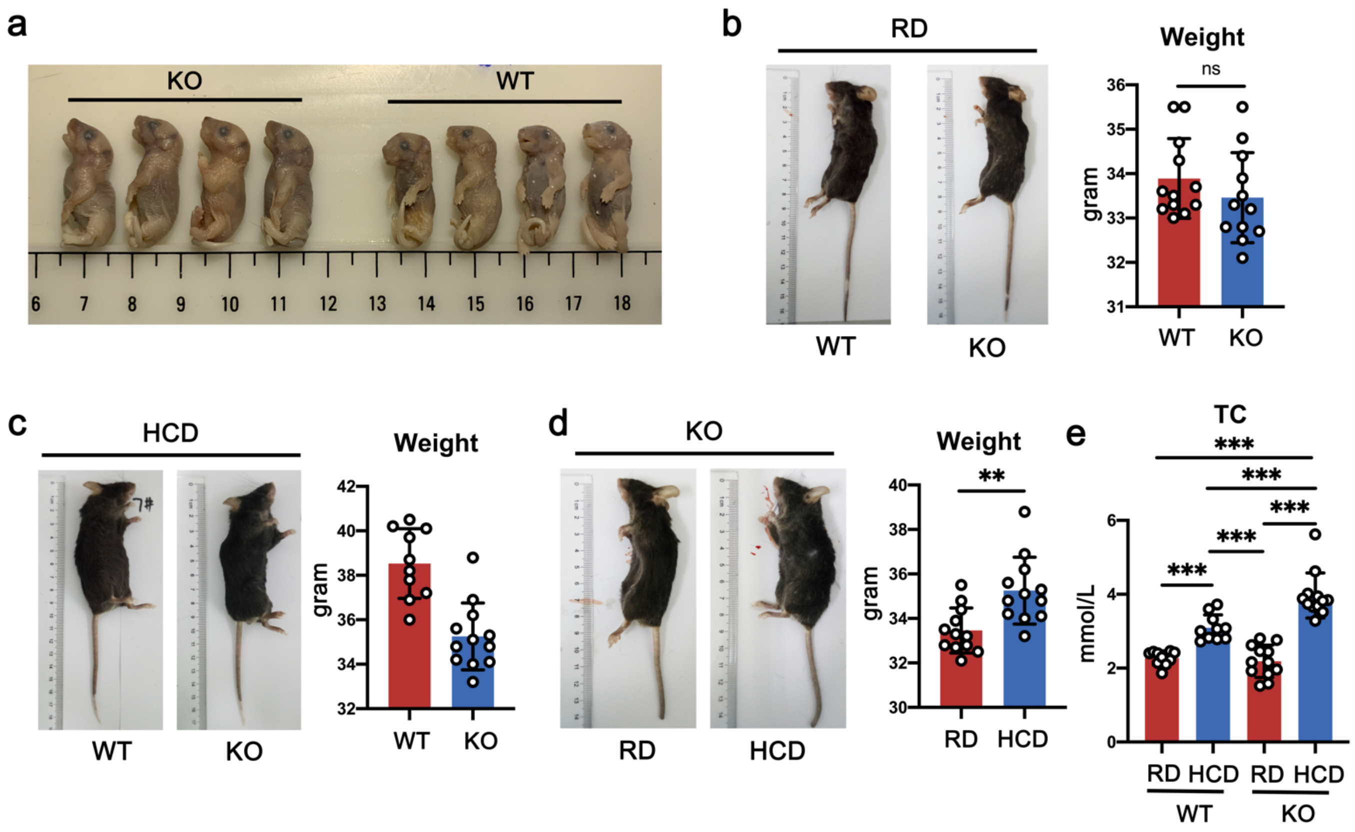

Supplementary figure 5 a There is no obvious disability and dysplasia in *Lrp3*$^{-/-}$ mice and no significant difference in the overall size compared with WT mice one day after birth. **b** Under regular diet (RD), adult (3 months) *Lrp3*$^{-/-}$ and WT mice have no significant difference in body weight (n = 12, two-tailed Student's *t*-test). **c** Under high-cholesterol diet (HCD), the weight of the WT mice is significantly greater than the weight of *Lrp3*$^{-/-}$ mice (n = 12, two-tailed Student's *t*-test). **d** Comparison of general view and body weight of *Lrp3*$^{-/-}$ mice before and after HCD (n = 12, two-tailed Student's *t*-test). **e** Comparison of serum TC of *Lrp3*$^{-/-}$ mice and WT mice before and after HCD (n = 12, one-way ANOVA). Data are shown as the mean ± SD. $^{*}P < 0.05$; $^{**}P < 0.01$; $^{***}P < 0.001$. n indicates the number of mice per group.

Supplementary figure 6

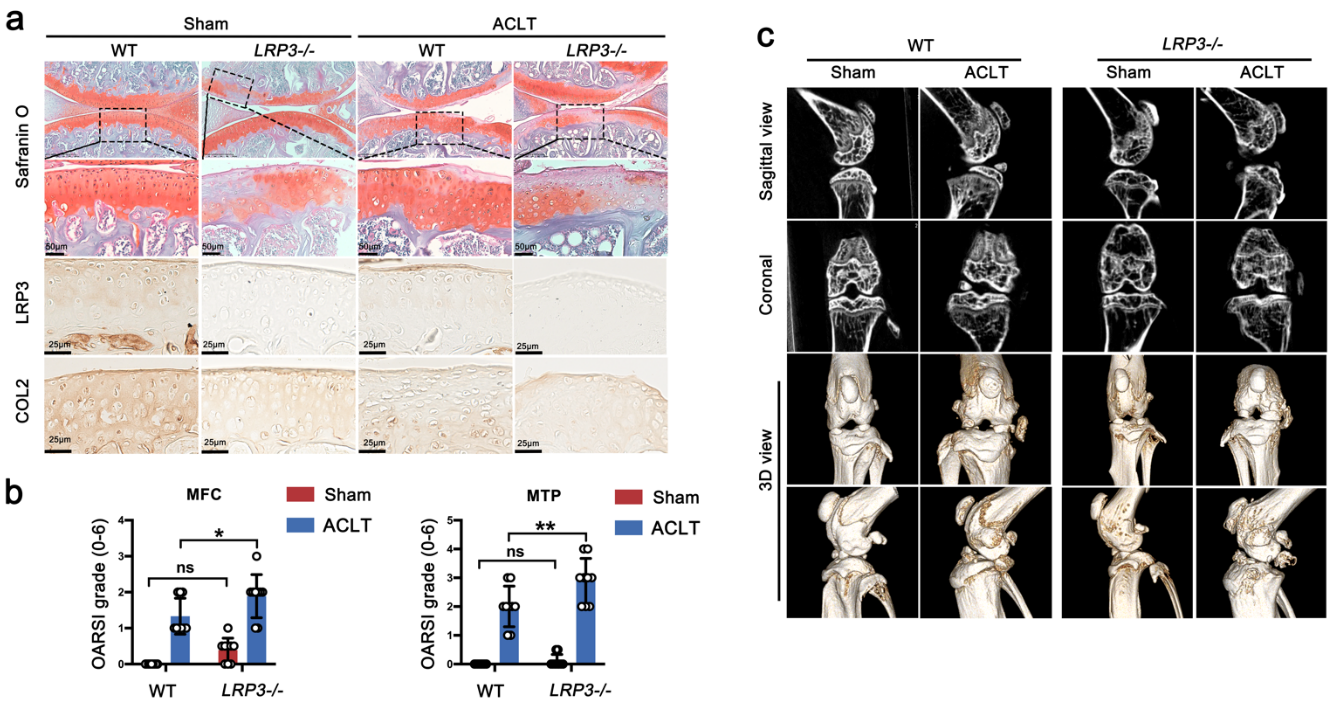

Supplementary figure 6 a Representative images of safranin O-fast green (n = 9), LRP3 and COL2 IHC staining (n = 4) of knee joints from wild type (WT) mice and *Lrp3*$^{-/-}$ mice subjected to Sham or ACLT operation for 4 weeks, insets indicate the regions shown in the enlarged images. **b** OARSI scores of WT mice and *Lrp3*$^{-/-}$ mice subjected to Sham or ACLT operation for 4 weeks (n = 9, one-way ANOVA). **c** Representative micro-CT images of WT mice and *Lrp3*$^{-/-}$ mice subjected to ACLT operation for 4 weeks (n = 4). Data are shown as the mean ± SD. **P* < 0.05; ***P* < 0.01; ****P* < 0.001. n indicates the number of mice per group.

Supplementary figure 7

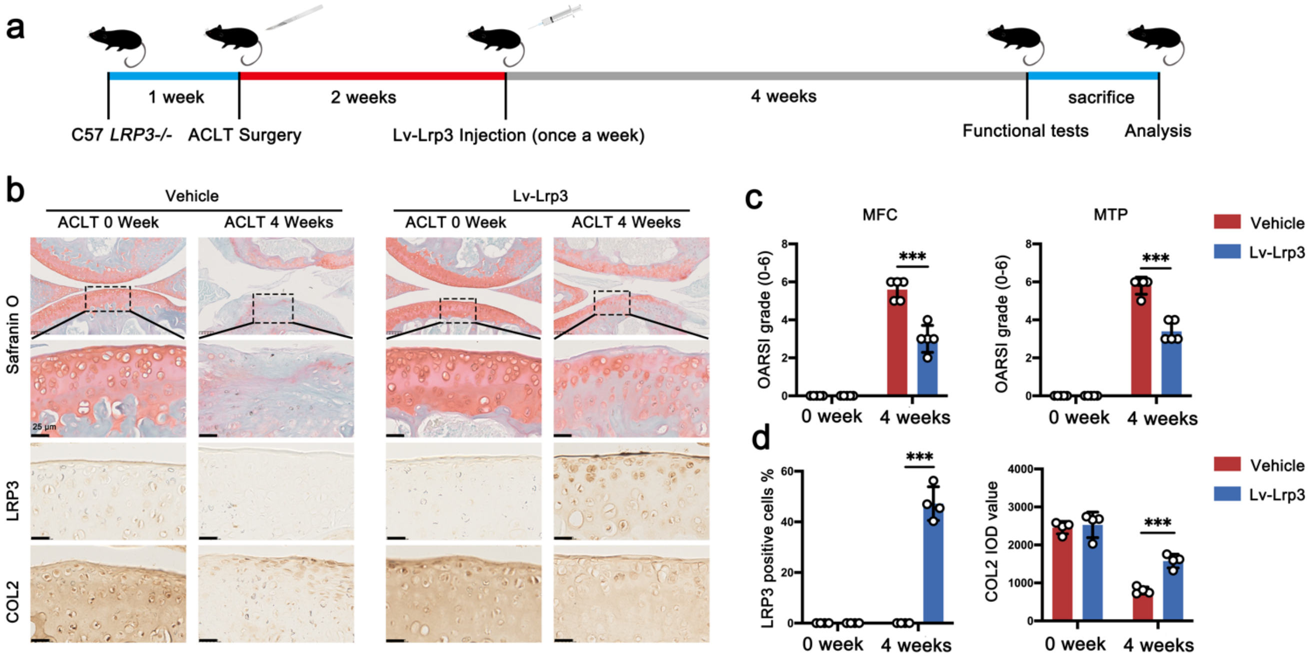

Supplementary figure 7 **a** Scheme of rescue experiments on *Lrp3*$^{-/-}$ mice after ACLT with Lv-Lrp3. **b** Representative images of safranin O-fast green (n = 5), LRP3 and COL2 IHC staining (n = 4) of knee joints from *Lrp3*$^{-/-}$ mice injected with Lv-Lrp3 or Lv-con335 for 4 weeks, insets indicate the regions shown in the enlarged images. **c** OARSI scores of *Lrp3*$^{-/-}$ mice injected with Lv-Lrp3 or Lv-con335 for 4 weeks (n = 5, two-tailed Student's *t*-test). **d** Quantification of LRP3-positive cells and IOD value for COL2 in *Lrp3*$^{-/-}$ mice injected with Lv-Lrp3 or Lv-con335 for 4 weeks (n = 4, two-tailed Student's *t*-test). Data are shown as the mean ± SD. $^{*}P < 0.05$; $^{**}P < 0.01$; $^{***}P < 0.001$. n indicates the number of mice per group.

Supplementary figure 8

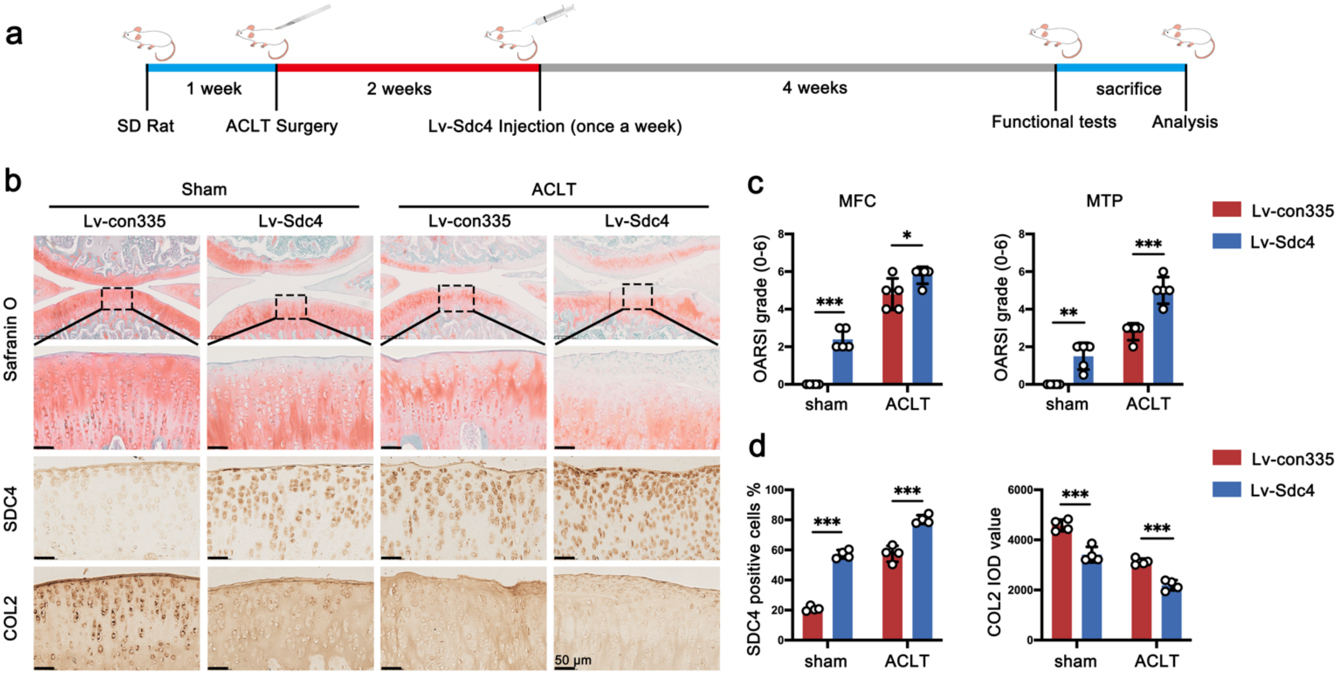

Supplementary figure 8 a Scheme of ACLT surgery and Lv-Sdc4 injection in rats. **b** Representative images of safranin O-fast green (n = 5), LRP3 and COL2 IHC staining (n = 4) of knee joints from Lv-Sdc4 injected rats and Lv-con335 injected rats subjected to Sham or ACLT operation for 4 weeks, insets indicate the regions shown in the enlarged images. **c** OARSI scores of Lv-Sdc4 injected rats and Lv-con335 injected rats subjected to Sham or ACLT operation for 4 weeks (n = 5, two-tailed Student's *t*-test). **d** Quantification of SDC4-positive cells and IOD value for COL2 in Lv-Sdc4 injected rats and Lv-con335 injected rats subjected to Sham or ACLT operation for 4 weeks (n = 4, two-tailed Student's *t*-test). Data are shown as the mean ± SD. $^{*}P < 0.05$; $^{**}P < 0.01$; $^{***}P < 0.001$. n indicates the number of mice per group.

Supplementary figure 9

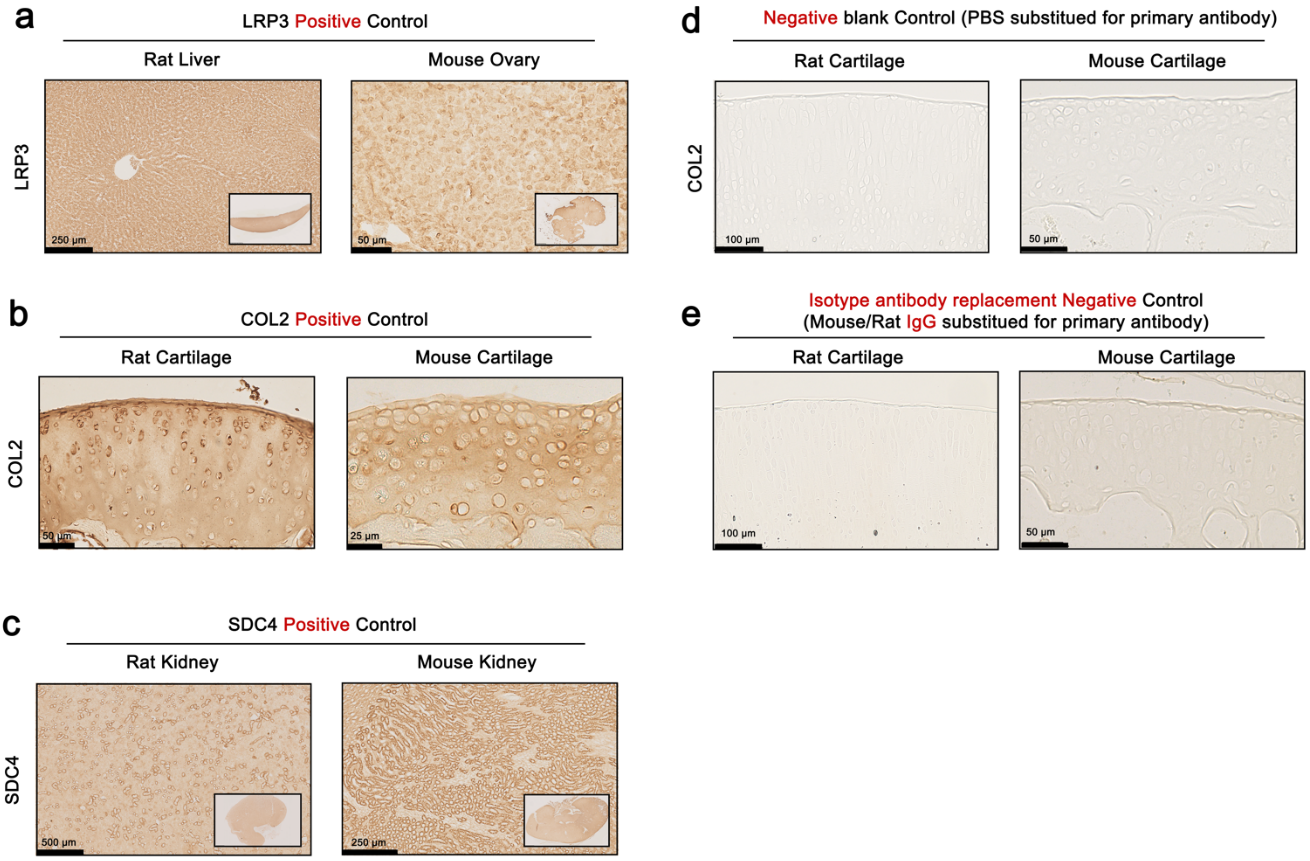

Supplementary figure 9 a LRP3 positive IHC staining in rat liver and mouse ovary (n = 3). **b** COL2 positive IHC staining in rat and mouse cartilage (n = 3). **c** SDC4 positive IHC staining in rat and mouse kidney (n = 3). **d** Negative blank control of IHC staining in rat and mouse cartilage (n = 3). **e** Isotype negative control of IHC staining in rat and mouse cartilage (n = 3). n indicates the number of biologically independent samples.

Supplementary Table 1. The clinical characteristics of patients

Meniscus injured patients (as control)							OA patients						
Gender	TC	LDL	BMI	Joint	Other diseases#	ICRS grade	Gender	TC	LDL	BMI	Joint	Other diseases#	ICRS grade
F	6.89	4.61	23.92	knee		IV	F	6.85	4.90	21.36	knee		IV
M	6.39	3.43	29.05	knee	DB, HT	III	F	6.63	4.27	22.86	knee	DB	IV
F	6.38	3.4	22.59	knee		II	F	6.53	4.12	26.67	knee	HT	IV
F	6.29	4.03	18.44	knee	HT	0	F	6.45	4.44	21.37	knee		IV
F	6.17	4.41	28.71	knee		IV	F	6.24	4.47	27.68	knee		IV
F	6.06	3.07	25.86	knee	DB	II	F	6.20	2.66	28.74	knee	HT	IV
M	5.97	2.37	19.20	knee		IV	F	6.18	4.05	27.34	knee	HT	IV
F	5.89	4.14	23.53	knee	DB, HT	II	F	6.16	3.16	27.59	knee	DB, HT	IV
F	5.87	4.14	20.70	knee		II	F	6.11	3.71	26.13	knee	DB, HT	IV
F	5.85	3.97	30.22	knee		IV	F	6.08	4.27	30.49	knee		IV
F	5.83	4.01	23.44	knee		IV	F	6.07	4.61	28.06	knee		IV
F	5.71	3.22	24.22	knee		IV	F	6.04	4.49	28.44	knee	HT	IV
M	5.71	4.02	24.46	knee		III	M	6.03	4.60	26.49	knee	HT	IV
F	5.68	3.93	26.78	knee	DB, HT	II	M	6.00	4.58	26.44	knee	HT	IV
M	5.68	3.88	22.32	knee	HT	II	M	5.97	4.37	27.40	knee		IV
F	5.66	3.39	19.92	knee	DB	III	F	5.93	4.18	28.04	knee		IV
F	5.64	3.78	21.56	knee		II	F	5.87	3.94	29.30	knee		IV
F	5.59	3.56	29.04	knee		II	F	5.87	3.83	27.48	knee	HT	IV
F	5.56	3.33	26.99	knee		0	F	5.86	3.90	31.64	knee	DB, HT	IV
F	5.55	3.47	21.22	knee		IV	F	5.84	3.85	29.39	knee		IV
F	5.53	3.9	22.43	knee		0	F	5.75	3.97	25.53	knee	DB, HT	IV
M	5.53	3.74	26.18	knee		II	M	5.74	3.90	29.04	knee		IV
F	5.48	3.15	23.96	knee		II	F	5.72	4.01	27.47	knee	DB, HT	IV
F	5.47	2.43	28.25	knee		IV	F	5.66	3.97	22.21	knee	DB, HT	IV
F	5.44	3.73	29.09	knee		III	F	5.63	3.24	27.03	knee		IV
F	5.36	3.68	27.25	knee		II	F	5.58	4.17	25.81	knee		IV
M	5.33	3.82	20.66	knee		II	F	5.56	3.61	24.62	knee	DB, HT	IV

M	5.32	3.29	27.73	knee	HT	III
F	5.29	3.47	28.80	knee		II
F	5.28	3.49	25.28	knee		II
F	5.26	3.09	25.72	knee	HT	II
M	5.23	3.84	21.63	knee	HT	II
F	5.23	2.59	19.36	knee		0
F	5.21	3.36	25.32	knee		III
F	5.20	1.68	17.44	knee		III
M	5.17	3.11	23.46	knee		II
M	5.14	3.96	26.45	knee		II
M	5.14	3.16	23.38	knee		I
M	5.12	3.38	23.60	knee		II
F	5.12	3.18	22.48	knee		II
M	5.11	3.1	23.72	knee	DB	II
F	5.10	3.17	19.82	knee		II
M	5.08	2.62	25.26	knee		III
F	5.07	3.43	18.92	knee		II
M	5.05	2.67	22.41	knee		I
F	5.04	3.28	25.39	knee		I
F	5.00	2.64	28.04	knee	DB, HT	I
F	4.96	3.05	27.48	knee		IV
F	4.93	2.48	20.69	knee		IV
F	4.89	3.43	21.50	knee		III
F	4.84	3.04	20.83	knee		II
F	4.79	4.58	25.15	knee		II
M	4.78	1.66	29.07	knee	HT	0
M	4.65	3.11	25.21	knee		I
F	4.64	3.08	21.23	knee		III
F	4.64	2.86	24.01	knee		I
F	4.60	3.18	19.71	knee		III
M	4.53	3.3	22.02	knee		0
M	5.53	3.75	26.78	knee	HT	IV
F	5.53	3.95	27.53	knee		IV
F	5.53	3.66	27.10	knee	HT	IV
F	5.53	3.77	26.91	knee		IV
F	5.50	3.08	28.28	knee	DB, HT	IV
F	5.49	4.12	25.34	knee		IV
F	5.47	3.32	24.35	knee		IV
F	5.45	3.92	20.70	knee		IV
M	5.45	3.98	29.40	knee	HT	IV
F	5.44	3.27	25.91	knee		IV
F	5.44	3.56	27.97	knee	HT	IV
F	5.42	3.62	26.99	knee	HT	IV
M	5.41	3.55	30.10	knee	DB, HT	IV
F	5.39	3.61	24.46	knee		IV
F	5.38	3.47	26.44	knee		IV
M	5.35	3.05	27.74	knee		IV
F	5.28	3.32	28.30	knee	DB	IV
F	5.28	3.75	29.52	knee		IV
F	5.23	3.79	25.85	knee		IV
F	5.18	3.37	25.33	knee		IV
F	5.14	3.25	28.88	knee	DB, HT	IV
F	5.13	3.12	27.24	knee	DB	IV
F	5.10	3.57	27.53	knee	DB, HT	IV
M	5.09	3.57	30.00	knee	DB, HT	IV
F	5.09	2.46	24.14	knee		IV
F	5.01	3.09	27.78	knee		IV
F	5.01	3.22	28.33	knee	DB, HT	IV
F	5.00	3.34	29.55	knee		IV
F	5.00	3.56	27.12	knee	DB	IV
M	4.99	3.57	28.73	knee	DB	IV
F	4.93	3.58	24.01	knee		IV

F	4.52	3.14	22.06	knee		0
M	4.47	3.3	22.23	knee		III
F	4.46	2.11	26.23	knee		I
F	4.43	2.98	20.66	knee		IV
F	4.39	3.93	21.79	knee	DB	II
F	4.39	2.69	22.55	knee		0
M	4.38	2.75	27.76	knee		II
F	4.37	2.68	26.90	knee		IV
F	4.23	2.31	22.21	knee		IV
M	4.22	2.55	28.73	knee	HT	II
M	4.13	2.49	28.37	knee		II
F	4.13	2.32	20.82	knee		II
F	4.10	2.57	19.15	knee		II
M	4.10	2.23	18.71	knee		II
M	4.06	2.67	22.91	knee	DB	I
M	4.01	2.5	29.06	knee		IV
M	3.91	2.11	24.31	knee		III
F	3.90	1.55	19.63	knee		III
M	3.87	1.99	22.99	knee		IV
M	3.86	1.97	22.15	knee	HT	IV
F	3.78	2.53	19.23	knee	HT	I
F	3.78	2.02	18.67	knee		I
F	3.70	2.08	23.31	knee		I
F	3.69	1.91	24.92	knee		IV
M	3.66	2.33	23.05	knee		II
M	3.59	1.99	24.09	knee		III
M	3.59	1.95	24.06	knee		0
F	3.49	1.95	20.17	knee		II
M	3.40	1.56	24.68	knee	HT	III
F	3.34	1.9	18.26	knee		0
M	3.30	2.34	29.76	knee		III
M	4.90	2.97	29.07	knee		IV
F	4.86	2.87	25.56	knee	DB	IV
F	4.83	3.15	29.86	knee	HT	IV
F	4.79	2.57	26.84	knee		IV
F	4.74	3.24	28.62	knee	HT	IV
M	4.73	3.41	28.38	knee	HT	IV
F	4.70	2.79	24.74	knee		IV
F	4.69	2.86	27.34	knee	DB	IV
M	4.64	2.88	25.99	knee	DB	IV
F	4.63	2.62	26.84	knee		IV
F	4.62	2.77	20.13	knee		IV
F	4.61	2.90	25.08	knee		IV
F	4.60	2.73	28.57	knee	DB	IV
M	4.57	3.51	26.57	knee		IV
F	4.52	3.04	27.12	knee		IV
M	4.52	3.01	28.33	knee	DB	IV
F	4.47	2.63	19.15	knee	DB, HT	IV
F	4.44	2.97	28.83	knee	DB	IV
M	4.42	2.70	28.71	knee	DB	IV
F	4.42	2.69	25.65	knee		IV
F	4.4	2.88	26.99	knee		IV
F	4.31	2.39	27.34	knee	HT	IV
F	4.30	2.18	20.57	knee		IV
F	4.27	2.56	27.29	knee	DB, HT	IV
F	4.26	2.64	22.72	knee	HT	IV
M	4.26	3.20	20.66	knee	DB	IV
F	4.26	2.89	27.93	knee	DB	IV
F	4.25	2.33	27.89	knee		IV
F	4.19	2.39	24.38	knee		IV
F	4.12	2.27	26.74	knee		IV
F	4.11	2.46	27.48	knee	DB, HT	IV

M	3.29	1.77	25.86	knee	DB, HT	III	F	4.10	2.71	24.24	knee		IV
M	3.28	1.81	29.41	knee		I	F	4.09	2.25	28.62	knee		IV
							M	4.07	2.80	29.03	knee	HT	IV
							F	4.02	2.44	25.65	knee	HT	IV
							F	4.02	2.54	26.22	knee	HT	IV
							F	4.01	2.07	22.66	knee		IV
							M	3.97	2.82	31.98	knee		IV
							F	3.90	2.16	22.06	knee	DB, HT	IV
							F	3.86	1.85	26.12	knee		IV
							F	3.84	2.22	23.05	knee	DB, HT	IV
							F	3.78	2.09	27.59	knee	DB, HT	IV

RA: rheumatoid arthritis, DB: diabetes, HT: hypertension

Supplementary Table 2. Primer sequences

Gene name		Sequences
Rn18s	F	5′- GTAACCCGTTGAACCCCATT -3′
	R	5′- CCATCCAATCGGTAGTAGCG -3′
Col2a1	F	5′- CACCGCTAACGTCCAGATGAC -3′
	R	5′- GGAAGGCGTGAGGTCTTCTGT -3′
Acan	F	5′- CATTCGCACGGGAGCAGCCA -3′
	R	5′- TGGGGTCCGTGGGCTCACAA -3′
Sox9	F	5′- TCCCCGCAACAGATCTCCTA -3′
	R	5′- AGCTGTGTGTAGACGGGTTG -3′
Mmp13	F	5′- CTGCGGTTCACTTTGAGGAC -3′
	R	5′- ACAGCATCTACTTTGTCGCC -3′
Adamts5	F	5′- CACGACCCTCAAGAACTTTTGC -3′
	R	5′- TCACATGAATGATGCCCACATAA -3′
Lrp1	F	5′- GGTCCTGAACTGAATCACGCCTTC-3′
	R	5′- TAGACACTGCCGCTCCGATACTC-3′
Lrp2	F	5′- CACCTCCTTCACCTGCGACAATC-3′
	R	5′- CATCCGAGCCATCCGAGCAATC-3′
Lrp3	F	5′- TGGGATGGCTGAACCACAGA -3′
	R	5′- CAGTGGGAAGTTAACAGCA -3′
Lrp4	F	5′- ACACGCTGCTACTGAACAACCTG-3′
	R	5′- GACACCACCTCCTCCACATTGC-3′
Lrp5	F	5′- CTTCATCCACCGTGCCAACCTG-3′
	R	5′- TCTGCCAGTCTGTCCAGTAGAGTG-3′
Lrp6	F	5′- GCCATCCGTCGCTCCTTCATTG-3′
	R	5′- GCCACCCAGTCAACAGCAATACC-3′
Sdc4	F	5′-TGTTGCTCCTCGGAGGTTTC-3′
	R	5′-GGAACCCGACAGCTCAAAGT-3′
