# Supplementary material for: Cholesterol-induced LRP3 downregulation promotes cartilage degeneration in osteoarthritis by targeting Syndecan-4
Source: Nat Commun. 2022 Nov 21;13:7139. doi: 10.1038/s41467-022-34830-4 (PMC9681739; doi:10.1038/s41467-022-34830-4)
Supplement: Supplementary file 3 — Reporting Summary [file 41467_2022_34830_MOESM3_ESM.pdf]

nature portfolio

Corresponding author(s): Yingfang Ao, Xiaoqing Hu, Jin Cheng

Last updated by author(s): Oct 9, 2022

Reporting Summary

Nature Portfolio wishes to improve the reproducibility of the work that we publish. This form provides structure for consistency and transparency in reporting. For further information on Nature Portfolio policies, see our Editorial Policies and the Editorial Policy Checklist.

Statistics

For all statistical analyses, confirm that the following items are present in the figure legend, table legend, main text, or Methods section.

n/a	Confirmed	
☐	☒	The exact sample size (*n*) for each experimental group/condition, given as a discrete number and unit of measurement
☐	☒	A statement on whether measurements were taken from distinct samples or whether the same sample was measured repeatedly
☐	☒	The statistical test(s) used AND whether they are one- or two-sided<br>*Only common tests should be described solely by name; describe more complex techniques in the Methods section.*
☐	☒	A description of all covariates tested
☐	☒	A description of any assumptions or corrections, such as tests of normality and adjustment for multiple comparisons
☐	☒	A full description of the statistical parameters including central tendency (e.g. means) or other basic estimates (e.g. regression coefficient) AND variation (e.g. standard deviation) or associated estimates of uncertainty (e.g. confidence intervals)
☐	☒	For null hypothesis testing, the test statistic (e.g. *F*, *t*, *r*) with confidence intervals, effect sizes, degrees of freedom and *P* value noted<br>*Give P values as exact values whenever suitable.*
☒	☐	For Bayesian analysis, information on the choice of priors and Markov chain Monte Carlo settings
☒	☐	For hierarchical and complex designs, identification of the appropriate level for tests and full reporting of outcomes
☒	☐	Estimates of effect sizes (e.g. Cohen's *d*, Pearson's *r*), indicating how they were calculated

Our web collection on statistics for biologists contains articles on many of the points above.

Software and code

Policy information about availability of computer code

Data collection	1) qRT-PCR analysis: Applied Biosystems StepOnePlus Real-Time PCR System (version. 2.3; Foster City, CA, USA);<br>2) Western blot: BIO-RAD ChemiDoc XRS+ system(version. 3.4.2);<br>3) Immunofluorescence analysis: confocal microscope (Olympus Life Science, Tokyo, Japan)<br>4) Hot plate test: hot plate meter (Ugo Basile SRL, Italy)<br>5) Weight bearing test: incapacitance tester (Ugo Basile SRL, Italy)<br>6) Nanoindentation analysis: situ nanomechanical test system (version. 2.0; TI-900 TriboIndenter, Hysitron, Minneapolis, MN, USA)<br>7) Micro-computed tomography (CT) analysis: micro-CT (Siemens Inveon MM Gantry, Berlin, Germany) and Inveon Research Workplace software (version. 3.0; Siemens Inveon MM Gantry, Berlin, Germany)<br>8) Statistical analysis: SPSS (version 20.0; IBM Corp, Chicago, IL, USA)
Data analysis	Analysis was performed using the SPSS 20.0 statistical software (IBM Corp) and Image analysis: ImageJ software (version v.1.51a, National Institutes of Health, MD, USA);

For manuscripts utilizing custom algorithms or software that are central to the research but not yet described in published literature, software must be made available to editors and reviewers. We strongly encourage code deposition in a community repository (e.g. GitHub). See the Nature Portfolio guidelines for submitting code & software for further information.

Data

Policy information about availability of data

All manuscripts must include a data availability statement. This statement should provide the following information, where applicable:

- Accession codes, unique identifiers, or web links for publicly available datasets
- A description of any restrictions on data availability
- For clinical datasets or third party data, please ensure that the statement adheres to our policy

All data generated or analyzed during this study are included in this published article (and its supplementary information files). The source data for the Figure1-7 and Supplementary figures generated in this study are provided in the Source Data file. The raw RNA sequencing data generated in this study have been deposited in the National Center for Biotechnology Sequences Read Archive under accession code ID PRJNA855888, [http://www.ncbi.nlm.nih.gov/bioproject/PRJNA855888]. Source data are provided with this paper.

Field-specific reporting

Please select the one below that is the best fit for your research. If you are not sure, read the appropriate sections before making your selection.

☒ Life sciences ☐ Behavioural & social sciences ☐ Ecological, evolutionary & environmental sciences

For a reference copy of the document with all sections, see nature.com/documents/nr-reporting-summary-flat.pdf

Life sciences study design

All studies must disclose on these points even when the disclosure is negative.

Sample size	Although no statistical methods were used to predetermined sample size in vitro and in vivo analyses, we conducted preliminary experiments to estimate variances in each assay and determined sufficient sample size.
Data exclusions	No samples or animals were excluded from analyses.
Replication	All experimental findings were reproduced independently at least four times. For each figure panel, the numbers of biologically independent samples, mice per group, or human specimens are indicated in the figure legends. Data shown in figure panels are the mean of all independent biological repeats.
Randomization	For in vitro experiments, cultures were randomly chosen for different treatments and experiments were performed multiple times.<br>For animal experiments, Lrp3-/- and WT littermates were allocated into groups based on sex, age, and<br>genotype. Male mice were selected for the experiment in order to avoid concerns about hormonal effect in female mice. After the selection, mice for the ACLT surgery, high cholesterol diet, or intra-articular injection were allocated randomly without subjective judgement.
Blinding	Cartilage destruction analysis, Hot plate test analysis, Weight bearing test analysis and immunohistochemistry<br>were performed by individuals who were blinded to the specific conditions of the experimental group.

Reporting for specific materials, systems and methods

We require information from authors about some types of materials, experimental systems and methods used in many studies. Here, indicate whether each material, system or method listed is relevant to your study. If you are not sure if a list item applies to your research, read the appropriate section before selecting a response.

Materials & experimental systems

n/a	Involved in the study	
☐	☒	Antibodies
☐	☒	Eukaryotic cell lines
☒	☐	Palaeontology and archaeology
☐	☒	Animals and other organisms
☐	☒	Human research participants
☒	☐	Clinical data
☒	☐	Dual use research of concern

Methods

n/a	Involved in the study	
☒	☐	ChIP-seq
☒	☐	Flow cytometry
☒	☐	MRI-based neuroimaging

Antibodies

Antibodies used	1) western blot analysis:<br>Proteins were analysed using antibodies against<br>COL2A1 (ab34712; Abcam, CA, USA; 1:1000)

GAPDH (TA-08; ZSGB-BIO, Beijing, China; 1:1000)
SOX9 (ab185966; Abcam, CA, USA; 1: 2000)
LRP3 (PA5-50051; Invitrogen, USA; 1:500)
SDC4 (ab74139; Abcam, CA, USA; 1:1000)
Ras (#3965; Cell Signaling Technology, MA, USA; 1:1000)
c-Raf (#9422; Cell Signaling Technology, MA, USA; 1:1000)
phospho-Erk1/2 (#4370; Cell Signaling Technology, MA, USA; 1:1000)
ERK1/2 (#4695; Cell Signaling Technology, MA, USA; 1:1000)
phospho-MEK1/2 (#9154; Cell Signaling Technology, MA, USA; 1:1000)
MEK1/2 (#9422; Cell Signaling Technology, MA, USA; 1:1000)
Adamts-5 (ab41037; Abcam, CA, USA; 1:1000).

Secondary antibodies:
Goat anti-Mouse IgG (ZB-2305, ZSGB-BIO, Beijing, China; 1:1000, HRP-conjuagted).
Goat anti-Rabbit IgG (ZB-2301, ZSGB-BIO, Beijing, China; 1:1000, HRP-conjuagted).

2) immunohistochemistry (IHC) assessment:
COL2A1 (ab34712; Abcam, CA, USA; 1:100)
LRP3 (human: ab115197; Abcam, CA, USA; 1:50) (rat/mouse: TA323023; Origene, USA; 1:50)
SDC4 (ab74139; Abcam, CA, USA; 1:50)
ARGSVIL (ab3773; Abcam, CA, USA; 1:50)

Secondary antibodies:
Goat anti-Mouse IgG (PV-6001, ZSGB-BIO, Beijing, China; 1:1000, HRP-conjuagted).
Goat anti-Rabbit IgG (PV-6002, ZSGB-BIO, Beijing, China; 1:1000, HRP-conjuagted).

3) Immunofluorescence (IF) analysis:
COL2A1 (ab34712; Abcam, CA, USA; 1:100)
SOX9 (ab185966; Abcam, CA, USA; 1:100)
LRP3 (sc-373736; Santa Cruz Biotechnology, USA; 1:50)

Secondary antibodies:
Goat anti-Rabbit IgG (A-11008, Thermo Fisher Scientific, USA; 1:200, Alexa Fluor™ 488)
Goat anti-Mouse IgG (A-11005, Thermo Fisher Scientific, USA; 1:200, Alexa Fluor™ 594)

Validation

All antibodies used in this study were validated by the suppliers as follows:
COL2A1 (ab34712; Abcam, CA, USA; 1:1000) for WB: Reacts with: Mouse, Rat, Sheep, Chicken, Cow, Dog, Human, Pig; Suitable for: ELISA, IP, ICC/IF, IHC-P, WB; manufacturer's website (https://www.abcam.cn/collagen-ii-antibody-ab34712.html)
GAPDH (TA-08; ZSGB-BIO, Beijing, China; 1:1000) for WB: Reacts with: Human, mouse, rat, monkey, dog; Suitable for: WB; manufacturer's website (http://www.zsbio.com/product/TA-08)
SOX9 (ab185966; Abcam, CA, USA; 1: 2000) for WB: Reacts with: Mouse, Rat, Human; Suitable for: Flow Cyt (Intra), ICC/IF, WB, IHC-P; manufacturer's website (https://www.abcam.cn/sox9-antibody-epr14335-78-ab185966.html)
LRP3 (PA5-50051; Invitrogen, USA; 1:500) for WB: Reacts with: Human, Mouse, Rat; Suitable for: WB; manufacturer's website (https://www.thermofisher.cn/cn/zh/antibody/product/LRP3-Antibody-Polyclonal/PA5-50051)
SDC4 (ab74139; Abcam, CA, USA; 1:1000) for WB: Reacts with: Human, Mouse, Rat; Suitable for: ELISA, WB, IHC-P, ICC/IF; manufacturer's website (https://www.abcam.cn/syndecan-4-antibody-ab74139.html)
Ras (#3965; Cell Signaling Technology, MA, USA; 1:1000) for WB: Reacts with: Human, Mouse, Rat, Monkey, D. melanogaster, Pig, S. cerevisiae; Suitable for: WB; manufacturer's website (https://www.cellsignal.com/products/primary-antibodies/ras-antibody/3965?site-search-type=Products&N=4294956287&Ntt=%233965&fromPage=plp&_requestid=1272211)
c-Raf (#9422; Cell Signaling Technology, MA, USA; 1:1000) for WB: Reacts with: Human, Mouse, Rat, Monkey; Suitable for: WB; manufacturer's website (https://www.cellsignal.com/products/primary-antibodies/c-raf-antibody/9422?site-search-type=Products&N=4294956287&Ntt=%239422&fromPage=plp&_requestid=1273100)
phospho-Erk1/2 (#4370; Cell Signaling Technology, MA, USA; 1:1000) for WB: Reacts with: Human, Mouse, Rat, Hamster, Monkey, Mink, D. melanogaster, Zebrafish, Bovine, Dog, Pig, S. cerevisiae; Suitable for: WB, IP, IHC, IF; manufacturer's website (https://www.cellsignal.com/products/primary-antibodies/phospho-p44-42-mapk-erk1-2-thr202-tyr204-d13-14-4e-xp-rabbit-mab/4370?site-search-type=Products&N=4294956287&Ntt=%234370&fromPage=plp&_requestid=1273519)
ERK1/2 (#4695; Cell Signaling Technology, MA, USA; 1:1000) for WB: Reacts with: Human, Mouse, Rat, Hamster, Monkey, Mink, D. melanogaster, Zebrafish, Bovine, Dog, Pig, C. elegans; Suitable for: WB, IP, IHC, IF; manufacturer's website (https://www.cellsignal.com/products/primary-antibodies/p44-42-mapk-erk1-2-137f5-rabbit-mab/4695?site-search-type=Products&N=4294956287&Ntt=%234695&fromPage=plp)
phospho-MEK1/2 (#9154; Cell Signaling Technology, MA, USA; 1:1000) for WB: Reacts with: Human, Mouse, Rat, Monkey; Suitable for: WB, IP; manufacturer's website (https://www.cellsignal.com/products/primary-antibodies/phospho-mek1-2-ser217-221-41g9-rabbit-mab/9154?site-search-type=Products&N=4294956287&Ntt=%239154&fromPage=plp&_requestid=1275777)
MEK1/2 (#9422; Cell Signaling Technology, MA, USA; 1:1000) for WB: Reacts with: Human, Mouse, Rat, Monkey; Suitable for:WB; manufacturer's website (https://www.cellsignal.com/products/primary-antibodies/c-raf-antibody/9422?site-search-type=Products&N=4294956287&Ntt=%239422&fromPage=plp&_requestid=1276178)
Adamts-5 (ab41037; Abcam, CA, USA; 1:1000) for WB: Reacts with: Mouse, Rat, Human
; Suitable for: WB; manufacturer's website (https://www.abcam.cn/adamts5-antibody-ab41037.html)
COL2A1 (ab34712; Abcam, CA, USA; 1:100) for IHC: Reacts with: Mouse, Rat, Sheep, Chicken, Cow, Dog, Human, Pig; Suitable for: ELISA, IP, ICC/IF, IHC-P, WB; manufacturer's website (https://www.abcam.cn/collagen-ii-antibody-ab34712.html)
LRP3 (human: ab115197; Abcam, CA, USA; 1:50) for IHC: Reacts with: Human; Suitable for: IHC-P, ELISA; manufacturer's website（https://www.abcam.cn/lrp3-antibody-ab115197.html）
LRP3 (rat/mouse: TA323023; Origene, USA; 1:50) for IHC: Reacts with: Human, Mouse, Rat; Suitable for: IHC-P; manufacturer's website (https://www.origene.com.cn/catalog/antibodies/primary-antibodies/ta323023/lrp3-rabbit-polyclonal-antibody)
SDC4 (ab74139; Abcam, CA, USA; 1:50) for IHC: Reacts with: Human, Mouse, Rat; Suitable for: ELISA, WB, IHC-P, ICC/IF;

manufacturer's website (https://www.abcam.cn/syndecan-4-antibody-ab74139.html)
ARGSVIL (ab3773; Abcam, CA, USA; 1:50) for IHC: Reacts with: Rat, Sheep, Rabbit, Horse, Guinea pig, Cow, Cat, Dog, Human, Pig; Suitable for: ICC/IF, ELISA, Sandwich ELISA, IHC-Fr, IHC-P, WB; manufacturer's website (https://www.abcam.cn/aggrecan-argxx-antibody-bc-3-ab3773.html)
COL2A1 (ab34712; Abcam, CA, USA; 1:1000) for IF: Reacts with: Mouse, Rat, Sheep, Chicken, Cow, Dog, Human, Pig; Suitable for: ELISA, IP, ICC/IF, IHC-P, WB; manufacturer's website (https://www.abcam.cn/collagen-ii-antibody-ab34712.html)
SOX9 (ab185966; Abcam, CA, USA; 1:100) for IF: Reacts with: Mouse, Rat, Human; Suitable for: Flow Cyt (Intra), ICC/IF, WB, IHC-P; manufacturer's website (https://www.abcam.cn/sox9-antibody-epr14335-78-ab185966.html)
LRP3 (sc-373736; Santa Cruz Biotechnology, USA; 1:50) for IF: Reacts with: Mouse, Rat, Human; Suitable for: WB, IP, IF, IHC(P), ELISA; manufacturer's website (https://www.scbt.com/zh/p/lrp3-antibody-d-8;jsessionid=wLHGALAxKO4dOEi9wObpJJnTUrlyPticRVhkDxmxeeVcA4iG_Zo5!489435925)

Eukaryotic cell lines

Policy information about cell lines

Cell line source(s)	HEK-293T cells were purchased from GeneChem Co. Ltd. (Shanghai, China).
Authentication	None of these cell lines were authenticated by us.
Mycoplasma contamination	We confirmed that cell lines used were negative for mycoplasma contamination.
Commonly misidentified lines (See ICLAC register)	No commonly misidentified cell lines were used.

Animals and other organisms

Policy information about studies involving animals; ARRIVE guidelines recommended for reporting animal research

Laboratory animals	8-week-old male C57BL/6 mice (WT and lrp3-/-) and 8-week-old male Sprague-Dawley(SD) rats were subjected to induction of experimental OA. A total of 90 C57BL/6-Lrp3 global knockout mice (Lrp3-/- mice) were purchased from Cyagen Biosciences, Inc. (Jiangsu, China) and were created by CRISPR/Cas9-mediated genome engineering. The housing conditions for mice is maintained in a SPF environment. The ambient temperature is maintained at 18-22 ℃, the relative humidity is maintained at 50% - 60%, and the average lighting time is 10-14 hours.
Wild animals	The study did not involve wild animals.
Field-collected samples	The study did not involve samples collected from the field.
Ethics oversight	Ethical approval (for rats and mice) was received from the Animal Care and Use Committee of Peking University Health Science Center. We have complied with all relevant ethical regulations for animal testing and research. Animal experiments were conducted in accordance with appropriate international guidelines.

Note that full information on the approval of the study protocol must also be provided in the manuscript.

Human research participants

Policy information about studies involving human research participants

Population characteristics	Human OA cartilage samples (n=21, 14 females and 7 males with mean age of 64.7 years) were excised from patients undergoing total knee replacement (TKA). Normal human cartilage samples (n=5, 2 females and 3 males with mean age of 53.3 years) were isolated from the knee joints of donors of trauma patients.
Recruitment	Patients with a osteoarthritis undergoing arthroplasty were recruited for offering the cartilage tissue. Human Ethics Committee of Peking University Third Hospital approved the use of these tissues, and written informed consent was obtained from all patients before the operative procedure.<br>The clinical data of the patients (n=191), including BMI, Total cholesterol (TC), Low-density lipoprotein (LDL) etc., were obtained from the Department of Sports Medicine, Peking University Third Hospital. All patients were from the Department of Sports Medicine, Peking University Third Hospital from January 2019 to March 2020. Recruitment bias is unlikely to impact this study as no patient comparisons are performed and no analysis of demographic or clinical covariates on cellular properties is performed.
Ethics oversight	This study was performed with the approval of the Human Ethics Committee of Peking University Third Hospital. We have complied with all relevant ethical regulations for work with human participants. And the patients' informed consent was obtained.

Note that full information on the approval of the study protocol must also be provided in the manuscript.
